# Phlagmasia Dolens, Puerperal Fever, and Mammary Abscess

**Published:** 1875-05

**Authors:** B. H. Washington


### PHLEGMASIA DOLENS, PUERPERAL FEVER, AND MAMMARY ABSCESS.

By B. H. WASHINGTON, M.D.

In looking over some numbers of the Atlanta Medical and Surgical Journal, I was much interested in the treatment proposed by Dr. J. M. Johnson for phlegmasia dolens; not interested in my own behalf, for ill consequences very rarely attend my obstetrical cases, but interested in behalf of suffering woman. It then occurred to me that, peradventure, her sufferings might be alleviated by making known the plans by which my own patients have suffered so little.

If the curious reader shall ask, why is it that you are not troubled with some of the ill consequences so frequently met with in obstetrical cases, my answer is very brief: it is mostly due to the fact that I dry-cup my white patients, (colored ones seldom requiring it). By means of dry-cupping there is no occluded os, for it relaxes the os and contracts the fundus, and there cannot therefore be any absorption of broken down coagula or of pus. Furthermore, the abraded surfaces all heal over in half the time necessary without dry-cupping. I have cured an ulcer on the arm by dry-cupping and a wet bandage in three weeks, on which another physician had exhausted his skill for seven months, and in about the same time an ulcer on a woman's nipple, on which a great many prescriptions had been tried, without effect, for four months.

The same powerful curative influence is exerted on the nerves of the uterus when dry-cupping is used, and the consequence is, that with ordinary care no one need fear any bad results from labors. I also use it as an excito-secretory agent to secure an early and free secretion of milk, and this it will insure. One patient whom I attended ten times, always had tender breasts, without milk, at first, but dry-cupping always relieved it, and frequently, when dry-cupped at night, the child could take the breast by midnight. To this treatment I attribute the fact that in my own practice I have never had a single breast to inflame. Perhaps it may be best for me to state that the dry-cupping is always tried on the spinal column, and with cups $2\frac{1}{4}$ to $2\frac{1}{2}$ inches in diameter; also, to secure a free secretion of milk, I apply a smaller cup on the neck. In my opinion, the fact that my patients are rarely troubled with any of the usual disagreeable consequences of confinement is due chiefly to dry-cupping.

About twenty-six or twenty-seven years ago I had a case of puerperal fever, and as the treatment was remarkably successful, though not laid down in the books, perhaps it may be well enough to mention it. The patient had been accidentally exposed to a strong night wind, and on the third day after delivery I was called in. Found her with a high, full and strong pulse, lochia suppressed, and excessive abdominal tenderness; so great that her clothes were removed from the abdomen, and she could not bear the weight of the sheet. A thin, tight, wet bandage was applied over the whole abdomen, not very cold at first, but gradually cooled, and changed by an attendant every minute and a half or two minutes. A pitcher of water was placed by the bedside, containing bicarb. potass. about thirty or forty grains to the pint, and she was allowed to drink freely. The following evening she was relieved sufficiently to be turned over on her side, and dry-cupped the whole length of the spine, and wet bandages continued. The lochial discharge was soon re-established; the wet bandages cured the inflammation, and the two visits were all that were necessary. Had she been treated according to the plans laid down in the books, half a dozen visits, and perhaps a consultation and a week's suffering, would probably have been the result.

Perhaps it may prove advantageous for me to mention another peculiarity of my management of puerperal cases. My preceptor, when he placed in my hands the first work on practice, said to me, "Make yourself acquainted with diseases and your remedial agencies, and then think for yourself, and never trouble yourself about any man's treatment;" and thus trained, I have felt myself privileged to vary sometimes from rules laid down in the books. I have for a long time made it a rule to deliver the placenta *within half an hour after the birth of the child, as it comes away much easier then than at any other time*, and for every hour's delay in its delivery the consequences become more disagreeable or injurious. This can readily be accomplished. While watching with my right hand for the pulsation in the cord to cease, I manipulate the uterus with the left hand, so as to cause it to contract, and, taken at this time the contraction can be easily secured. As soon as the child is disposed of I take the cord a turn or two around my fingers, and manipulating the uterus properly with my left hand, a steady gentle traction is made on the cord, and thus managing have often delivered the placenta in fifteen minutes after the birth of the child, and not longer than half an hour at any time. As soon as the placenta has descended sufficiently low for the membranous portion at the junction of the cord to be felt, it is best to bend the first joint of the forefinger at right angles, so as to make a hook, and after that there is not a particle of trouble, and the uterus contracts firmly without hæmorrhage. When the placenta is delivered, a bandage with a suitable compress is applied, so as to prevent any possibility of uterine relaxation, and if white the patient is dry-cupped the whole length of the spine, and an opiate administered, and a quiet night with full breasts in the morning are the pleasing results. Of course it would not be proper for anybody to pull on the umbilical cord; for he might, as some midwives do, invert the uterus, or produce fatal hæmorrhage; but the plan suggested above, if judiciously carried out, will most assuredly save a vast amount of suffering.

When I first commenced practising, the rules of the books were followed, with intense anxiety on my part and very disagreeable consequences to the patient; but I found that every hour's delay of the delivery of the placenta after the birth of the child made matters worse, and my practice was changed, until the placenta was delivered within the first half hour. In my first enthusiasm after my discovery that dry-cupping would hasten tedious labors, by relaxing the os uteri and external parts, and at the same time produce contractions of the fundus uteri, I relied upon dry-cupping to hasten the delivery of the placenta, but I found that the above plan was far the best, and relied upon dry-cupping for hastening labors, and this object it accomplishes most admirably. Prof. E. D. Fenner reported a case in labor thirty hours, and he had decided to use instruments, when he recollected my article recommending dry-cupping; tried it, and safely delivered the woman in thirty minutes.

It may appear impertinent for me to be using and suggesting plans not in accordance with standard authorities, but as long as I find my patients better off by not following their advice than by following it, I do not feel under any legal or moral obligation to follow their plans, and especially do I feel absolved from all such obligations when the sufferings of women may be alleviated.

